# Exploring the chemotherapeutic potential of currently used kinase inhibitors: An update

**DOI:** 10.3389/fphar.2022.1064472

**Published:** 2023-01-09

**Authors:** Rajashri R. Naik, Ashok K. Shakya

**Affiliations:** ^1^ Faculty of Allied Medical Sciences, Pharmacological and Diagnostic Research Center, Al-Ahliyya Amman University, Amman, Jordan; ^2^ Faculty of Pharmacy, Pharmacological and Diagnostic Research Center, Al-Ahliyya Amman University, Amman, Jordan

**Keywords:** FDA, cancer kinase inhibitors, BCR-ABL, EGFR, BRAF, MEK, ALK, BTK inhibitor

## Abstract

Protein kinases are enzymes that transfer phosphate to protein, resulting in the modification of the protein. The human genome encodes approximately 538 kinases. Kinases play a role in maintaining a number of cellular processes, including control of the cell cycle, metabolism, survival, and differentiation. Protein kinase dysregulation causes several diseases, and it has been shown that numerous kinases are deregulated in cancer. The oncogenic potential of these kinases is increased by a number of processes, including overexpression, relocation, fusion point mutations, and the disruption of upstream signaling. Understanding of the mechanism or role played by kinases has led to the development of a large number of kinase inhibitors with promising clinical benefits. In this review, we discuss FDA-approved kinase inhibitors and their mechanism, clinical benefits, and side effects, as well as the challenges of overcoming some of their side effects and future prospects for new kinase inhibitor discovery.

## Introduction

Kinases are enzymes that transfer the phosphate group from ATP to a specific substrate during phosphorylation. Depending on the nature of the chemical, the kinases involved may be protein kinases, lipid kinases, carbohydrate kinases, nucleophosphate kinases, or nucleoside diphosphate kinases. The phosphorylatable sites in eukaryotes include serine (ser) threonine (Thr), and tyrosine (tyr) (Adam and Hunter, 2018). Other amino acids that are phosphorylatable include arginine (Arg), Lysine (Lys), and cysteine (Cys) (Ciesla et al., 2011). Phosphorylation alters the substrate’s functionality by controlling the signaling pathway through cellular location, amplification, or interactions with regulatory proteins. In the human genome, protein kinases account for approximately 2% of the genetic material. These kinases are further classified into groups, families, and subfamilies (Manning et al., 2002a; Manning et al., 2002b). More than 500 protein kinases have been identified and their conformational changes and structure have been identified using x-ray crystallography. Protein kinases play a vital role in various cellular functions, such as metabolism, cell cycle regulation, cell survival, and cell differentiation. The target proteins undergo conformational changes as a result of the kinases’ activation, which include the phosphorylation of substrates (amino acids) such as serine, threonine, and tyrosine (Johnson and Lewis, 2001). The process of phosphorylation by kinase on the substrate (target proteins) is a controlled process, and any changes in the regulatory processes leads to disease. There are several mechanisms that lead to kinase dysregulation, such as overexpression, fusion point mutation, and dysregulation of signaling pathway, and these all increase the oncogenic capabilities of the kinases. Deregulation can lead to altered expression of a kinase and change its function, as well as affect its initiation and survival. One example of deregulation is BRAF, a proto-oncogene mutant that encodes serine/threonine protein kinase B-Raf and is found in 40%–50% of melanoma cases (Dankner et al., 2018). V600E is the mutation in BRAF that makes MAPK (mitogen-activated protein kinase) constitutively active, resulting in enhanced cell proliferation of the cell (Śmiech et al., 2020). Epidermal Growth Factor Receptor (EGFR) is another oncogene that belongs to the ErbB family of tyrosine kinases. Mutation in EGFR and overexpression of EGFR protein leads to the dysregulation of signals that favor tumor cells in terms of proliferation, survival, and metastasis (Thomas and Weihua, 2019).

## Kinase inhibitors for cancer treatment

The development of small molecules to target kinases has significantly increased due to the success of imatinib, the first tyrosine kinase inhibitor, which targeted the Bcr-Abl receptor. Since its discovery 20 years ago, large numbers of small-molecule kinase inhibitors have been developed to treat cancer. It was the first tyrosine kinase inhibitor to be approved by the US FDA (in 2001) for treating chronic myelogenous leukemia. In this review, we will discuss the different types of KI, examples of FDA-approved kinase inhibitors, some of the clinical trials that led to their approval, their side effects, and their limitations. Finally, we will emphasize how some of these limitations can be overcome by understanding the mechanism that leads to resistance.

## Types of kinase inhibitors in cancer therapy

There are many kinase inhibitors (Tables 1–Tables 3), which depending on their mode of target binding and mechanism of action can be divided into seven types. The different types of inhibitor, along with an example and mode of action, are summarized in Table 4.

**TABLE 1:** Chemical structures of tyrosine kinase inhibitors used in chemotherapy.

Name (alphabetical order)	Chemical structure
Afatinib	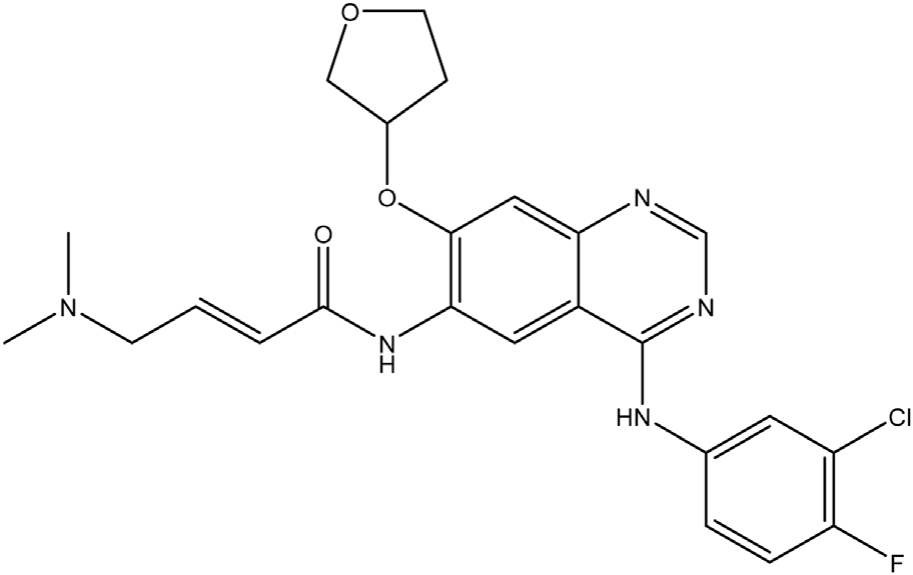
Axitinib	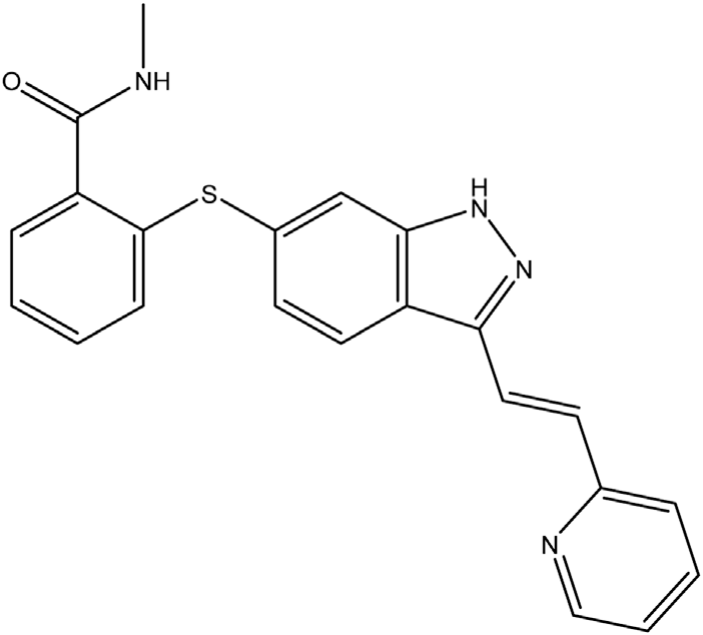
Bosutinib	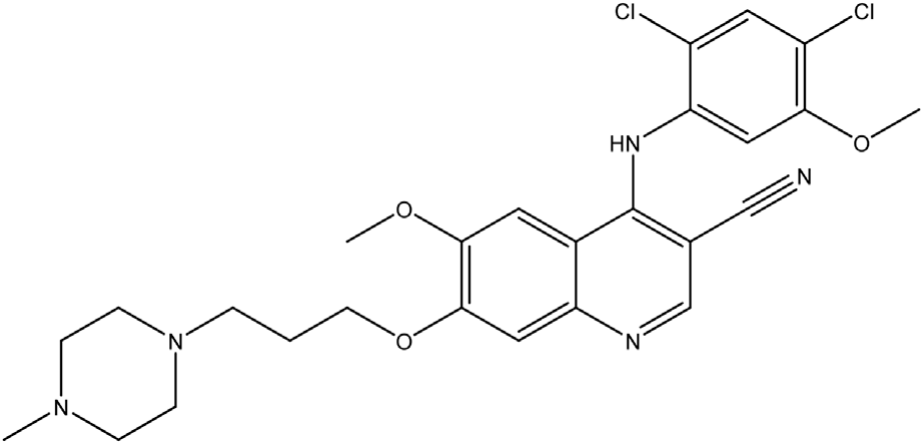
Cobimetinib	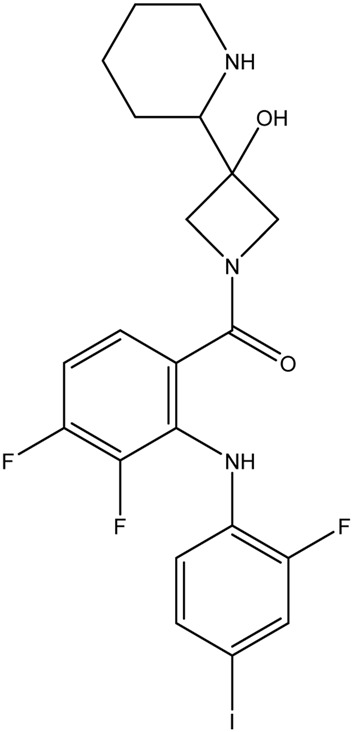
Crizotinib	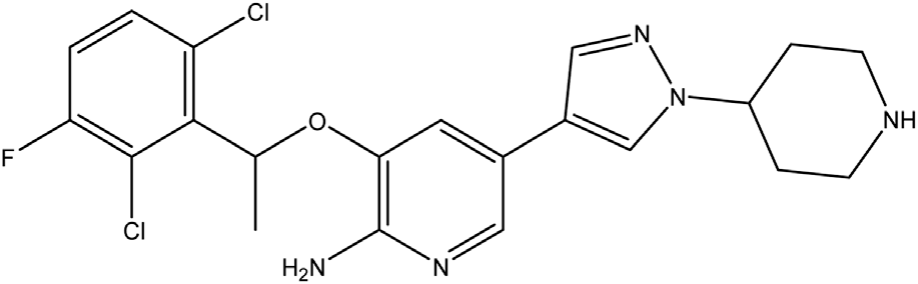
Dacomitinib	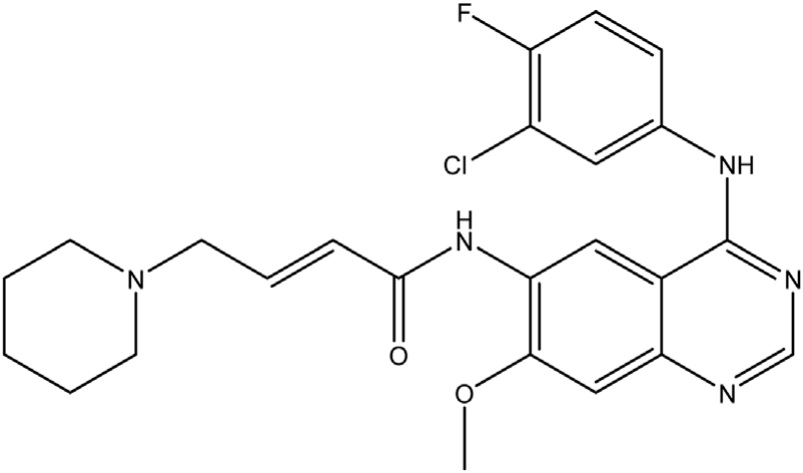
Dasatinib	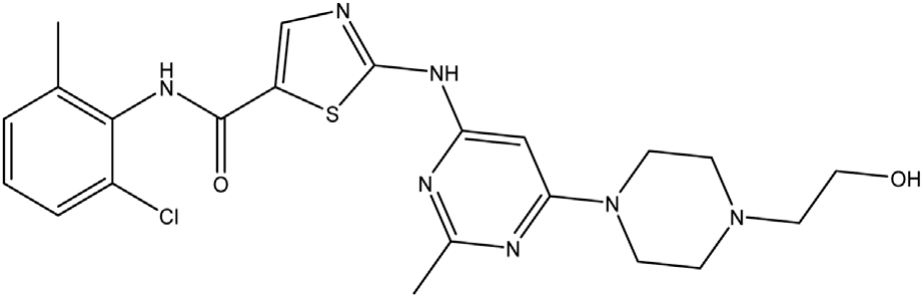
Erlotinib	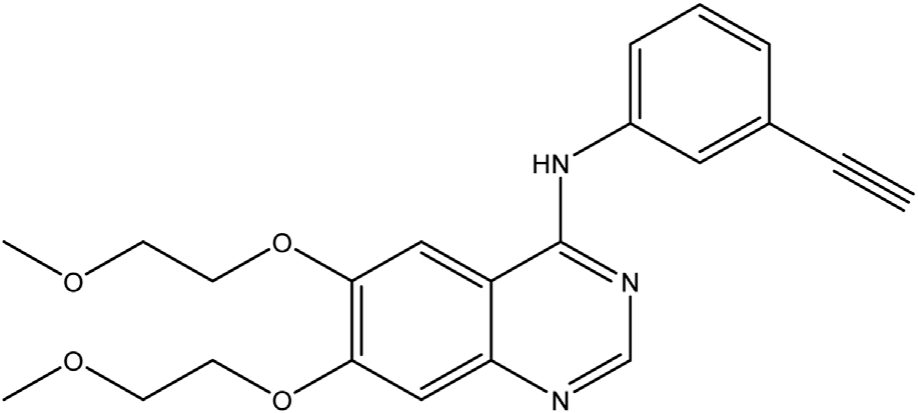
Everolimus	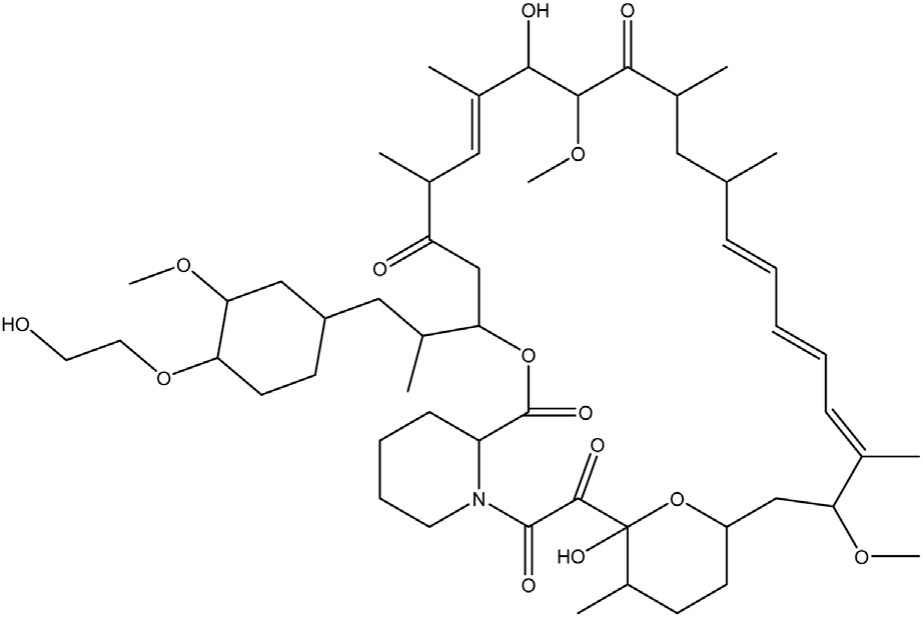
Gefitinib	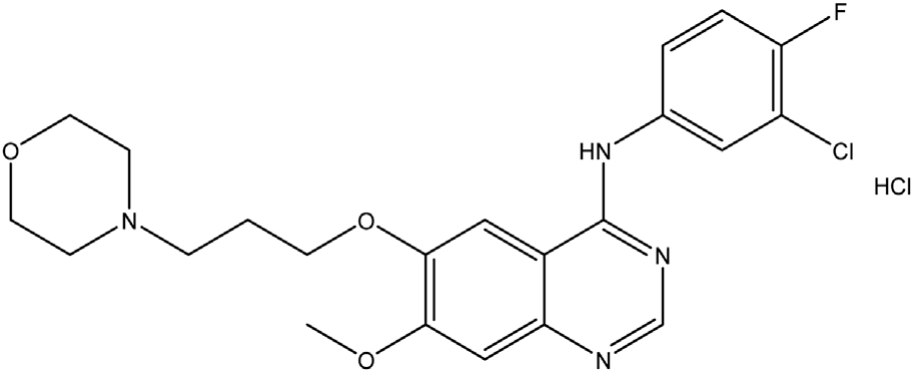
Ibrutinib	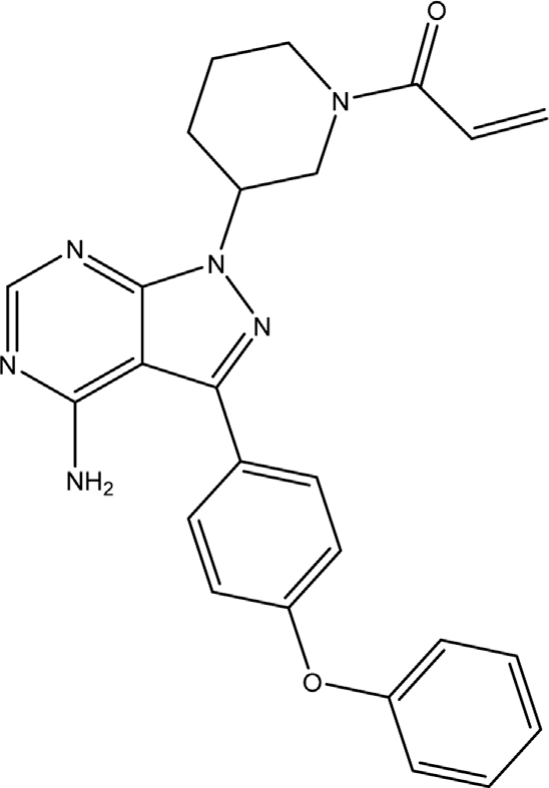
Imatinib	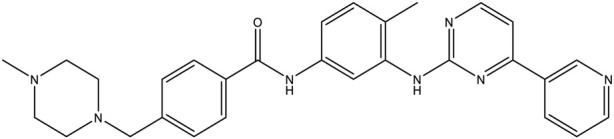
Lenvatinib	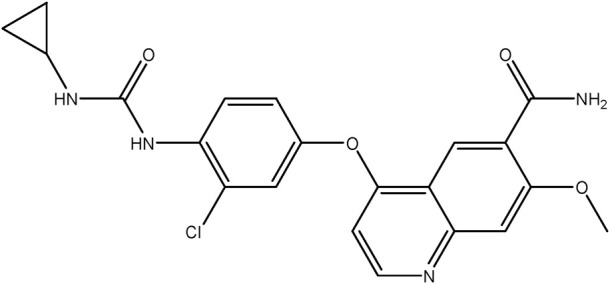
Neratinib	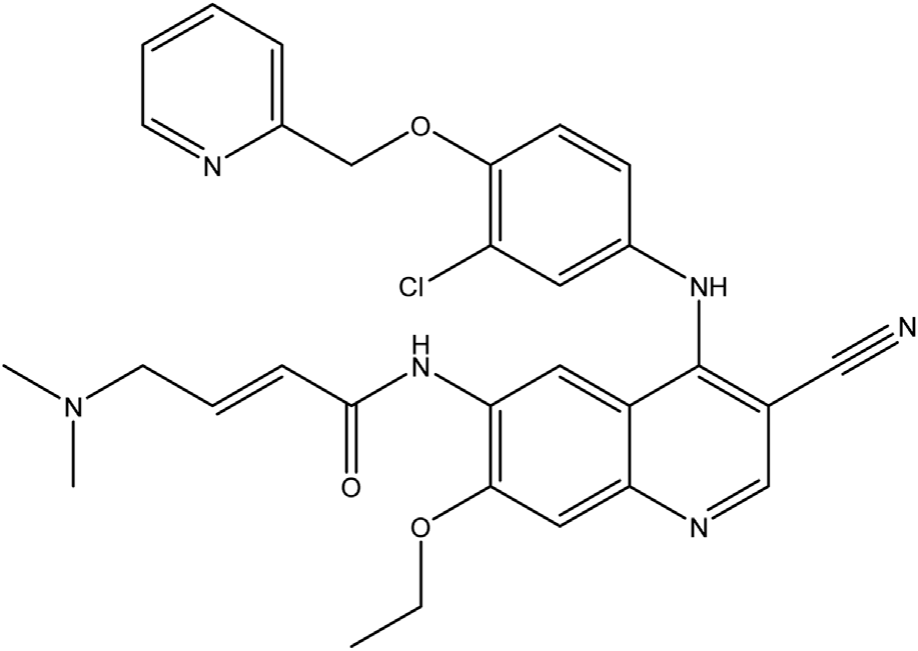
Nilotinib	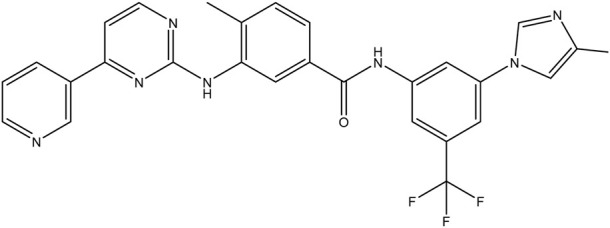
Pazopanib	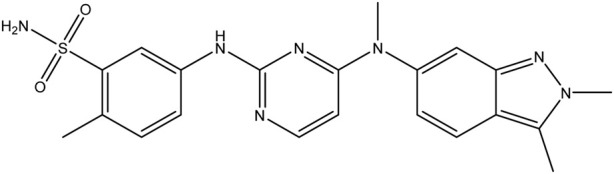
Ponatinib	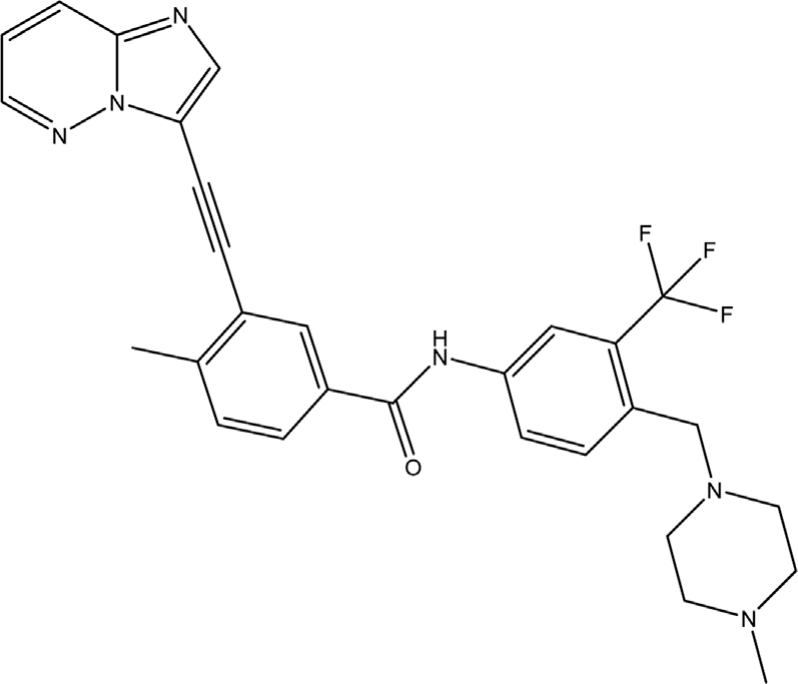
Ruxolitinib	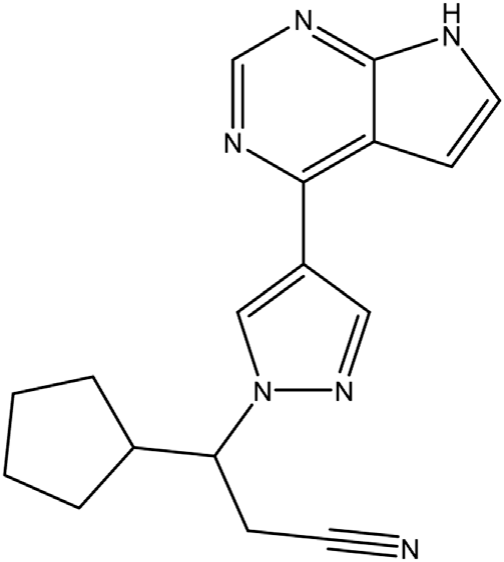
Sorafenib	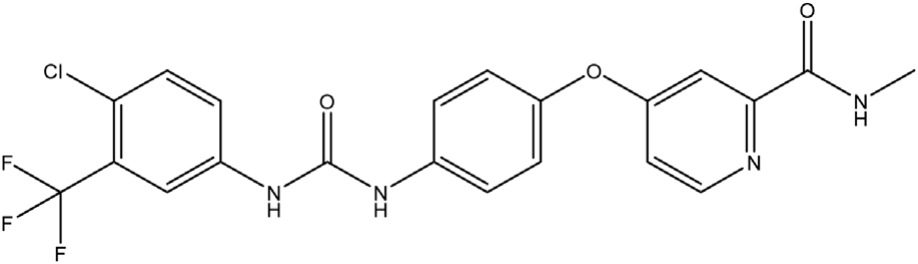
Sunitinib	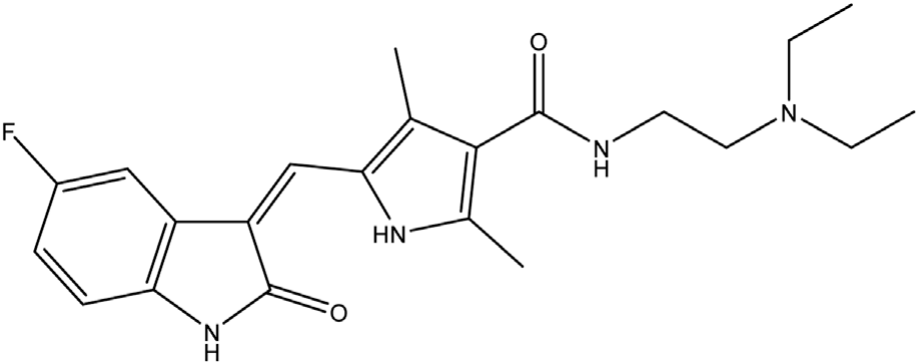
Tacrolimus	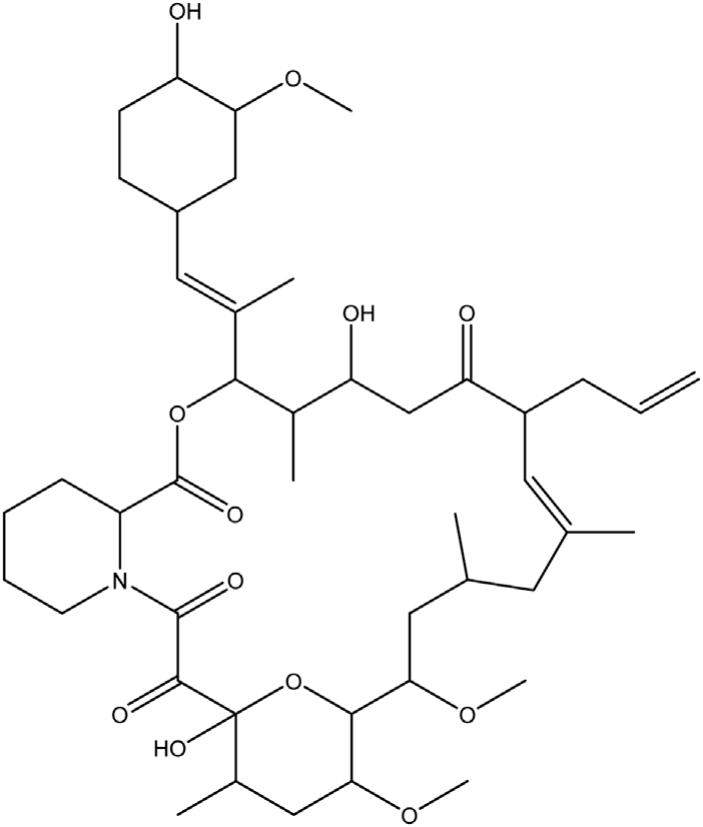
Temsirolimus	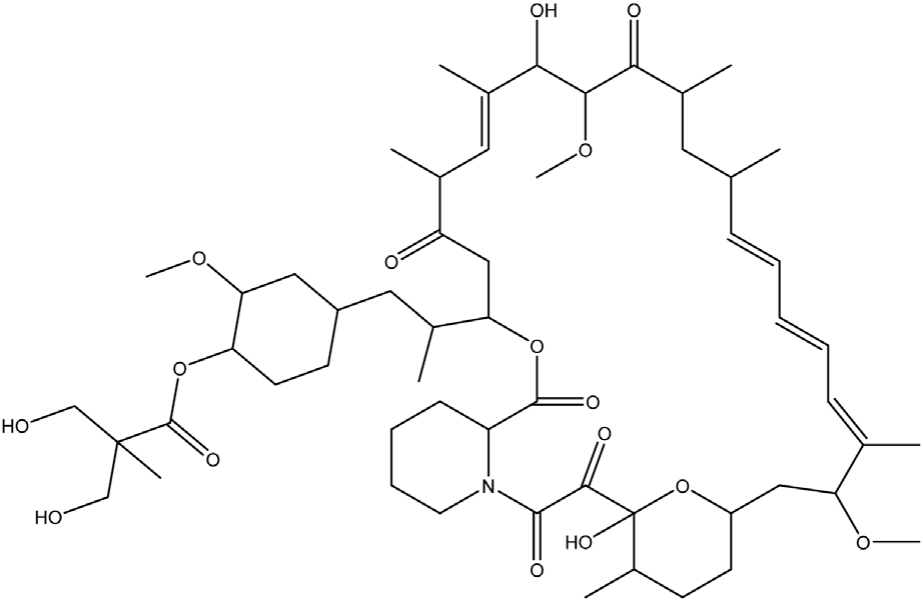
Trametinib	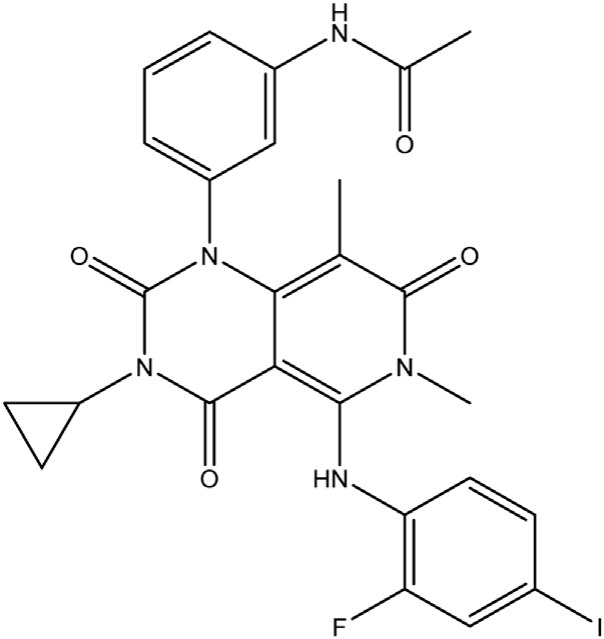
Vemurafenib	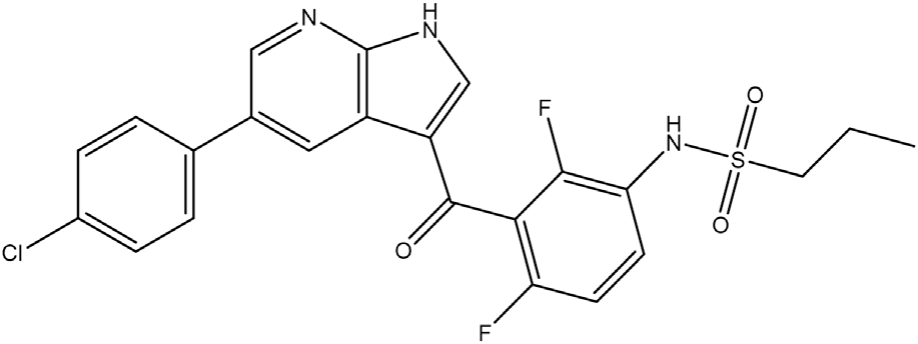

**TABLE 2:** Therapeutic uses of FDA-approved kinase inhibitors.

S.N.	Name and type	Inhibitor	Therapeutic use	FDA approval	References
1	Dasatinib type I	Bcr-Abl, c-Kit, PDGFRA B, ephrin, Src kinases	Ph-positive CML. It is resistant to the other Bcr-Abl inhibitors	FDA approved	Kantarjian et al. (2009)
2	Bosutinib type 1	Src –Abl kinase	Newly diagnosed CML	FDA December 2017	Cortes et al. (2018)
3	Crizotinib	c met, ALK tyrosine kinase	EML4-ALK fusion gene-positive NSCLC and ROS1 gene rearrangement-positive NSCLC	FDA approved 2011	Lim et al. (2017)
				FDA approved 2016	
4	Gefitinib	EFGRs	Against non-small cell lung cancer	FDA approved 2003	Molina et al. (2008)
5	Erlotinib	EFGRs	Against non-small cell lung cancer	FDA approved 2004	Molina et al. (2008)
6	Sunitinib	VEGFR, VEGFR2, PDGFRβ, KIT, RET, CSF1R, and FLT3	Imatinib-resistant GIST, adjuvant therapy in adult patients at high risk of RCC following nephrectomy	FDA approved 2006	
7	Lapatinib	EGFR, HER2 TKI	Patients with a combination of HER2-positive advanced and metastatic breast cancer with letrozole as a first-line agent, treatment of patients with HER2-positive advanced breast cancer	FDA approved 2007	Cameron and Stein, (2008)
				FDA approved 2010	
8	Pazopanib	VEGFR, PDGFR (A and B), c-Kit, and FGFR	Metastatic RCC and treatment of advanced-stage soft tissue sarcoma	FDA approved 2009	Sternberg et al. (2010)
				FDA approved 2012	
9	Ruxolitinib	JAK2, Janus kinase family (a non-receptor TK)	For intermediate to high-risk MF. Hydroxyurea-resistant or -intolerant PV; intermediate to high risk of MK; hydroxyurea-resistant or -intolerant PV	FDA approved 2011	(James et al. (2005), Kralovics et al., (2005)
				FDA approved 2014	
10	Vemurafenib	BRAF kinase inhibitor	For patients with BRAF mutant V600E metastatic melanoma	FDA approved 2011	Chapman et al. (2011)
11	Imatinib type II	Bcr-Abl kinase	As the first line therapy in the treatment of chronic CML. Rare hematologic malignancies and proto-oncogene c-Kit- or tyrosine-protein kinase kit (c-Kit)-mutated GIST	FDA approved	Druker et al. (2001), Kantarjian et al. (2012)
				FDA approved 2008	
12	Nilotinib	Bcr-Abl kinase inhibitor	As a front-line therapy for patients with chronic-stage CML and in patients who have developed resistance or are intolerant to imatinib	FDA approved	Weisberg et al. (2005)
13	Sorafenib	VEGFR TKIs, VEGFRs, PDGFRs, and FLT3R	For the treatment of metastatic renal cell carcinoma. Second-line agent for treating patients with metastatic RCC. For HCC. For metastatic thyroid cancer	FDA approved 2005, 2007, 2013	Escudier et al. (2007), Escudier et al. (2009), Brose et al. (2014)
14	Ponatinib	Binds to T315I mutant kinases	For patients with chronic-phase myeloid leukemia who are resistant or intolerant to at least two prior kinase inhibitors	FDA in 2014 narrowed down the recommended use for adult patients with T315I-positive CML or ALL patients	Lipton et al. (2016), Nicolini et al. (2017)
15	Trametinib Type III	MEK inhibitor	For the treatment of BRAF-mutated V600E metastatic melanoma. Combination of trametinib with dabrafenib as a first-line therapy. BARF V600E-mutated metastatic melanoma	FDA approved 2013	Odogwu et al. (2018)
				FDA approved 2014	
16	Cobimetinib	Inhibitor of MEK	Combination of vemurafenib (BRAF inhibitor) and MEK inhibitor cobimetinib for treating patients with mutant BRAF V600E metastatic melanoma	FDA approved	Eroglu and Ribas, (2016)
17	Afatinib type VI	EGFR TK	Used as a first-line therapy in patients with NSCLC. Second-line therapy in patients with advanced squamous cell carcinoma	FDA approved	Soria et al. (2015), Wind et al. (2017)
		ErbB group			
		HER 2, HER4			
		T790M mutation of EGFR, and wild-type EGFR			
18	Dacomitinib	EGFR kinase inhibitor	As the first-line therapy for patients with metastatic NSCLC and EGFR exon 19 deletion (Del19) or exon 21 L852R substitution mutation	FDA approved 27 September 2018	Reckamp et al. (2014); Wu et al. (2017b)
19	Neratinib	EGFR tyrosine kinase	As an adjuvant therapy in patients with early-stage HER2-positive breast cancer	FDA approved	Martin et al. (2017)

**TABLE 3:** Mechanism of action, results of clinical trials, and side effects of kinase inhibitors.

S.No.	Name	Route of administration and MOA	Clinical trial	Side effects	References
1	Dasatinib	Oral, binds to active conformation of the kinase Bcr-Abl	Multiple phase III trial. Effective cytogenic response		Conchon et al. (2011)
2	Bosutinib	Oral	Multinational phase III (BFORE). Higher efficiency in molecular and cytogenic response	Diarrhea results in leftover level of liver enzymes. Cardiovascular toxicity uncommon	Cortes et al. (2018), Brummendorf et al. (2022)
3	Crizotinib	Oral	Open-label phase II trial—significant increase in the median PFS of 7.7 months (HR on progression death 49% and response rate 65%)	Vision disorders, nausea, vomiting, diarrhea, constipation, and elevated liver enzymes	Shaw et al. (2013), Solomon et al. (2014)
			Open-label phase III trial—significantly improved median PFS of 10.9 months		
4	Gefitinib	Oral competitive antagonist that binds to the ATP site of EFGRs	Clinical trial—progression-free survival 10.8 months; survival mean 30.5 months	Acne rash, nausea, diarrhea, anorexia, stomatitis, and dehydration	Forsythe and Faulkner, (2004), Nakamura et al. (2018)
5	Erlotinib	Oral competitive antagonist that binds to the ATP site of EFGRs	PFS 13.1 months; overall survival 24.68 months	High rates of severe skin rashes, diarrhea, nausea, and vomiting stomatitis	(Zhou et al. (2011a), Xiong et al. (2019)
6	Sunitinib	Oral multi-target ATP competitive TKIs	phase III randomized trial—disease-free survival of 16.8 years	Grade 3 neutropenia, leukopenia, diarrhea, fatigue, nausea, hypertension, and hand-foot mouth syndrome	Demetri et al. (2006), Motzer et al. (2007), Ravaud et al. (2016)
			Landmark phase III trial—significantly longer PFS (11 months)		
7	Lapatinib	Inhibits the activity of HER2 TK in HER2-positive breast cancer patients	Phase III randomized open-label study—median time to progression significantly increased to 8.4 months	Diarrhea (68%) and rash (46%). The cardiac side effect observed with trastuzumab was not observed with letrozole	Geyer et al., (2006), Schwartzberg et al. (2010)
			Double-blind controlled randomized phase III trial—significantly prolonged PFS, overall response rate of 28%, clinical benefits at 48%		
8	Pazopanib	Oral multi TK inhibitors	Placebo-controlled randomized phase III trial—PFS of 9.2 months	Diarrhea, change in hair color, and increase in liver enzymes	Sternberg et al. (2010), Escudier et al. (2014)
			Double-blind phase III trial (PISCES)—70% preferred pazopanib		
9	Ruxolitinib	JAK2	Phase III trials—discontinuation rate was 30% and 50% for 3 and 5 years, respectively	Anemia, thrombocytopenia, and herpes zoster infection	Harrison et al. (2012), Verstovsek et al. (2012), Vannucchi et al. (2015)
10	Vemurafenib	BRAF inhibitor	Landmark phase III randomized BRIM-3—OS increased significantly by 84%, median PFS of 5.3 months		Chapman et al. (2011)
11	Imatinib	Kinase inhibitors against PDGFR	Phase III international randomized study (IRIS)—complete hematologic response of 95.3%, cytogenic response 85.2%	Neutropenia, thrombocytopenia, anemia, elevated liver enzymes, and other drug-related adverse events	O'Brien et al. (2003); Druker et al. (2006)
12	Nilotinib	Bcr-Abl kinase inhibitor	Phase III multicenter randomized clinical trial (ENESTnd)—a high twofold response rate increase (44%), cytogenic response of 80%	Rashes, headache, diarrhea, retention of fluid, cardiovascular events, cytopenia, and biochemical abnormalities	Saglio et al. (2010), Kantarjian et al. (2011), Hochhaus et al. (2016)
			24-month twofold increase of 71%, cytogenic response of 26%		
13	Sorafenib	VEGFR	Phase III randomized trial (TARGET)—median PFS of 5.5 months overall survival	Hypertension, fatigue, anemia, vomiting, and diarrhea	Escudier et al. (2007), Escudier et al. (2009), Brose et al. (2014)
			Multicenter phase III trial of sorafenib (SHARP)—significantly increased the median OS (10.7 months)		
			Phase III multicenter randomized placebo-controlled trial (DECISION)—PFS of 10.8 months		
14	Ponatinib	Binds to T315I mutant kinases	Phase III trial (EPIC)—owing to arterial embolism in an earlier report, the use of ponatinib was not established	Rash (47%), abdominal pain (46%), dry skin (42%), thrombocytopenia (46%) headache (43%), constipation (41%), diarrhea, pyrexia, myocardial infarction, anemia, neutropenia, and pancytopenia. Owing to arterial embolism, the FDA narrowed down its use in 2014	Lipton et al. (2016), Nicolini et al. (2017)
			Philadelphia Positive Acute Lymphoblastic Leukemia and Chronic Myeloid leukemia Evaluation Trial (PACE)—significantly longer OSR at 24 months (84%)		
15	Trametinib	Inhibitor of MEK	Phase III randomized clinical trial (METRIC)—significantly increased median PFS of 4.8 months	Rash, diarrhea, and peripheral edema. Secondary skin neoplasm was not observed with a combination of MEK inhibitors, but was observed with BRAF kinase inhibitor	Lugowska et al. (2015), Long et al. (2017b), Dummer et al. (2020)
			COMBI-d trial—median PFS of 9.3 months, OS was 25.1 months		
			Another trial (COMBI-v)- OS and media PFS of 12 months of 72%		
			Phase III control randomized trial (COMBI-AD)- 3 years relapse free survival rate 58%		
16	Cobimetinib	Inhibitor of MEK	Phase III randomized control trial comparison study—prolonged median PFS of 9.9 months	Pyrexia and elevated levels of liver enzymes	Larkin et al. (2014), Long et al. (2017a)
17	Afatinib	Irreversible ERBB	In patients for whom chemotherapy failed significantly increased PFS by 18%, and OS improved by 19%	Diarrhea, skin rash/acne	Paz-Ares et al. (2017)
			Phase III randomized clinical trial (LUX-Lung 8)	and fatigue	
18	Dacomitinib	Inhibits ERBB family members irreversibly, covalently binds to the receptor, and blocks the downstream signaling pathway	Dacomitinib is used to treat non-small cell lung cancer that has spread (metastatic) with an epidermal growth factor receptor (EGFR) exon 19 deletion or exon 21 L858R substitution mutation gene	Dose-limiting toxicity in the skin and gastrointestinal tract because they inhibit wild-type EGFRs	Sullivan and Planchard, (2016)
19	Neratinib	Inhibits EGFR tyrosine kinase	HER2-positive breast cancer	Diarrhea, nausea, and vomiting	Martin et al. (2017)

Results featured in the table are from clinical trials of the inhibitors only.

**TABLE 4:** Targets and mechanism of action of different types of FDA-approved tyrosine kinase inhibitors.

S.N.	Types of inhibitor	Targets	Mechanism of action	Examples of FDA-approved drugs	References
1	Type I	Binds to the active site in the kinase at the active conformation in the ATP (DFG-Asp in, αC-helix in)	Alters the active conformation and delays phosphorylation	Bosutinib, crizotinib, dasatinib, erlotinib, gefitinib, lapatinib, pazopanib, ruxolitinib, sunitinib, and vemurafenib	Roskoski, (2016)
2	Type II	DGF out confirmation, enzyme inactive form. Occupies part of the adenine-binding pocket and forms hydrogen bonds with the hinge region, connecting the small and large lobes of the enzyme	Interacts reversibly with targets by forming a hydrogen bond with the lipophilic hinge region. Interacts with the DFG out confirmation	Imatinib, sorafenib, axitinib, nilotinib, ponatinib, and Sunitinib	Roskoski, (2016)
3	Type III	Site adjacent to the ATP-binding pocket and mediates kinase activity	Binds to a specific enzyme, causing conformational change, which results in blocking the function of the kinase	Trametinib and cobimetinib	Roskoski, (2016)
4	Type IV	Targets outside the binding site without overlapping type III inhibitors	Do not bind to the binding site	Everolimus, sirolimus, temsirolimus	Gumireddy et al. (2005)
5	Type V	Bivalent inhibitor that binds to two different regions of the protein kinase domain	Irreversibly binds at the active site of the kinase	Lenvatinib	Okamoto et al. (2015)
6	Type VI	Binds to their target kinase	Interaction between the electrophilic groups of the inhibitors with nucleophilic cysteine	Afatinib, dacomitinib, and neratinib	Roskoski, (2016)
7	Type VII	Targets the extracellular domain of tyrosine kinase inhibitor		SSR128129E and WRG-28	Herbert et al. (2013), Grither and Longmore, (2018)

### Type I kinase inhibitors

Type I inhibitors bind to the active conformation of the kinase in the ATP (DFG-IN). This binding can alter the active conformation and delay phosphorylation. Currently, there are 10 FDA-approved tyrosine kinase inhibitors: bosutinib, crizotinib, dasatinib, erlotinib, gefitinib, lapatinib, pazopanib, ruxolitinib, sunitinib, and vemurafenib.

### Type II kinase inhibitors

Type II inhibitors target the DFG-out conformation, the inactive form of the enzyme (Kufareva and Abagyan, 2008). They interact reversibly with the targets by forming a hydrogen bond with the lipophilic hinge region. The high specificity of type II inhibitors is due to their lipophilic interactions, which contribute to the decreased toxicity of type II inhibitors compared with type I inhibitors (Bhullar et al., 2018). Imatinib, sorafenib, axitinib, nilotinib, ponatinib, and sunitinib are examples of type II kinase inhibitors.

### Type III or allosteric inhibitors

Type III inhibitors bind to the site adjacent to the ATP-binding pocket and mediate kinase activity (Amato et al., 2014). They are non-competitive inhibitors and bind to a specific kinase and exhibit high specificity. Binding to a specific enzyme causes conformational change that blocks the function of kinase. Type III inhibitors are classified into two subtypes: Type IIIA inhibitors bind to the adenine-binding site next to the ATP-binding site, and type IIIB inhibitors bind to other sites, then the site elsewhere. MEK1/2 are well-known type III inhibitors that bind to the cavity adjacent to the binding site of ATP. FDA-approved type III kinase inhibitors include trametinib and cobimetinib (Fasano et al., 2014).

### Type IV inhibitors or substrate-directed inhibitors

This type of inhibitor binds sites that are far removed from the ATP-binding site without overlapping with type III inhibitors. Everolimus, sirolimus, and temsirolimus are all examples of FDA-approved type IV kinase inhibitors (Gumireddy et al., 2005).

### Type V inhibitors

Type V inhibitors are bivalent and bind to the active site of the kinase irreversibly. These types of inhibitors are generally specific and potent.

### Type VI inhibitors

This type of inhibitor binds to their target kinase through interaction between the electrophilic groups of the inhibitors with a nucleophilic cysteine. FDA-approved drugs that belong to this group include afatinib, dacomitinib, and neratinib, which all target EGFR (Roskoski, 2019). Neratinib is a recently approved kinase inhibitor that inhibits human epidermal growth factor receptor two and prevents the recurrence of early stage HER2-positive breast cancer in patients (Rabindran et al., 2004).

### Type VII inhibitor

These types of inhibitors are defined as non-classical allosteric inhibitors that target the extracellular domain of tyrosine kinase inhibitors. They do not directly block the binding of the kinase domain/ligand-polypeptide site and are smaller than other inhibitors. Type VII inhibitors include SSR128129E (Herbert et al., 2013), which targets the fibroblast growth factor receptor, and WRG-28, which inhibits discoidin domain receptors (DDRs) (Grither and Longmore, 2018).

### Type I inhibitors

#### Dasatinib

Since the discovery of imatinib, several TKIs have been developed to inhibit Bcr-Abl kinases. These second generation TKIs were found to be more effective in treating patients whose treatment with imatinib failed or are resistant to imatinib (Shah et al., 2004). They are more potent at inhibiting Bcr-Abl than imatinib and nilotinib. Along with Bcr-Abl, c-Kit, PDGFRA, and B (the ephrin kinases), dasatinib also inhibits Src-kinase, which is associated with imatinib resistance. Dasatininb binds to the active conformation of the kinase Bcr-Abl. *In vitro* studies have shown that it can inhibit the activity of 14 out of 15 Bcr-Abl kinases, except for the mutant kinase T315I; therefore, it should be more efficient than imatinib. It may also be more efficient against imatinib-resistant CML (Shah et al., 2004). Dasatinib is approved by the FDA for treating Ph-positive CML, which is resistant to other Bcr-Abl inhibitors developed earlier. Various phase III clinical trials have shown a beneficial effect of dasatinib in CML patients who were resistant or intolerant to imatinib. Dasatinib and imatinib have been compared as first-line treatments (Kantarjian et al., 2009). Dasatinib was shown to have superior cytogenic responses to nilotinib, less efficient transformation to rapid or blast phase transformation, and higher PFS and OS rates (Kantarjian et al., 2009).

#### Bosutinib (Bosulif, SKI606)

Bosutinib is an orally administered third generation tyrosine kinase inhibitor. It is an Src-Abl kinase inhibitor like dasatinib but is more potent in inhibiting Bcr-Abl than imatinib. However, it does not have the potency to inhibit the activity of c-Kit or PDGFR kinase. The side effects of imatinib are caused by its inhibitory activity of c-Kit and PDGFR. A third generation TKI called bosutinib was designed as a result and has the same effectiveness as imatinib but with lesser side effects and a better safety profile than earlier Bcr-Abl inhibitors (Golas et al., 2003; Kantarjian et al., 2012). Bosutinib was efficacious at treating patients who were resistant to imatinib or intolerant to imatinib in phase I and II clinical trials (Kantarjian et al., 2014).

Bosutinib and imatinib were compared for their efficacy in treating newly diagnosed CML patients in a multinational phase III trial of the bosutinib-line in first-line chronic myelogenous leukemia treatment (B-FORE). Over 12 months, bosutinib exhibited higher efficiency with respect to the rate of molecular response (47.2% in the bosutinib group vs 39.9% in the imatinib group, *p* = 0.02). Similar results were observed in the complete cytogenic response (77.2% in the bosutinib group vs. 66.4% in the imatinib group, *p* = 0.0078). Based on the encouraging results of this trial, in December 2017, the FDA approved bosutinib for the treatment of newly diagnosed CML (Brümmendorf et al., 2015; Cortes et al., 2018).

Bosutinib has been found to be superior to other tyrosine kinase inhibitors because of its distinct side effects. The commonly encountered side effect with bosutinib is diarrhea, which can be addressed by a supportive measure, and leftover liver enzymes. Additionally, it is noteworthy that cardiovascular toxicity is rare with bosutinib (Brümmendorf et al., 2015; Cortes et al., 2018).

#### Crizotinib

Crizotinib was originally developed to inhibit c-Met. It also inhibits ALK tyrosine kinase activity and has been shown to be effective in NCSLC patients with an ALK fusion gene (Kwon and Meagher, 2012). Crizotinib has a beneficial effect on ROS-1 gene rearrangement, resulting in positive NSCLC due to its sequence similarity to the AKL and c-ros oncogene (ROS1) (Lim et al., 2017). Crizotinib was authorized by the FDA in 2011 for NSCLC with the EML4-ALK fusion gene, and again in 2016 for NSCLC with the ROS1 gene rearrangement.

In an open-label phase II trial, crizotinib was tested against pemetrexed in patients with NSCLC positive for the EML4-ALK fusion gene. A significant increase was observed in the median PFS in the crizotinib group (7.7 months) compared with pemetrexed (3 months). The HR on progression death with crizotinib was 49% and the response rate was 65%, compared with 20% for pemetrexed (Shaw et al., 2013).

Criozotinib was compared with chemotherapy (pemetrex plus cisplatin or carboplatin) in an open-label phase III randomized clinical trial in patients with ALK-positive NSCLC (profile 1,014). Crizotinib significantly improved the median PFS (10.9 months) compared with chemotherapy (7 months), and it had a risk of progression or death of 0.45 (CI 0.35–0.60, *p* < 0.001) (Solomon et al., 2014). This kinase inhibitor can cause vision problems, nausea, vomiting, diarrhea, constipation, and elevated liver enzyme levels (Shaw et al., 2013).

#### Gefitinib

Gefitinib and erlotinib are competitive antagonists that bind to the ATP site of EGFRs and are first-generation EGFR-TKIs. Both drugs are used against non-small cell lung cancer, which accounts for 80% of lung cancers (Molina et al., 2008). In May 2003 and November 2004, the FDA approved gefitinib and erlotinib, respectively. Many studies are being conducted to examine the reuse of these drugs in cancers that involve the activation of EGFRs.

These TKIs are well-established treatments for NSCLC and are used as a first-line treatment for patients with EGFR-mutated NSCLC (Sebastian et al., 2014). EGFR mutation is common in the Asian population, especially in female non-smokers and non-smokers in general (Tseng et al., 2017; Graham et al., 2018). Anti-apoptotic properties and uncontrolled cell growth are all associated with EGFR mutation. Adenocarcinoma is the most common type of NSCLS and the most common type of EGFR-mutated NSCLS. Gefitinib was initially approved for treating NSCLS but was later withdrawn from the market due to a lack of evidence that it had a beneficial effect on survival rate in unselected patients. However, it was approved once again, as the mutation is key to the response of gefitinib. Gefitinib is significantly more efficacious against NSCLC than chemotherapy; the progression-free survival rate was 10.8 months vs. 5.4 months, respectively, and the survival mean was 30.5 months vs. 23.6 months (Maemondo et al., 2010), respectively. In combination with chemotherapy, gefitinib was superior to gefitinib monotherapy (Nakamura et al., 2018).

Erlotinib was significantly superior to chemotherapy in advanced EGFR-positive NSCLC (PFS 13.1 vs. 4.6 months, respectively) (Zhou et al., 2011a), but its overall survival was lower than chemotherapy (24.68 vs. 26.16 months, respectively); it was superior to chemotherapy compared with chemotherapy with enhanced PS but not OS. A meta-analysis of gefitinib and erlotinib revealed that the efficacy of these two TKIs was comparable, although erlotinib caused more adverse effects.

Although they have not yet received therapy approval, gefitinib and erlotinib are being tested for their effectiveness against different malignancies. Although gefitinib was approved in 2005 for the treatment of pancreatic cancer in combination with chemotherapy, much of the research is still at the preclinical stage and the results are not promising. Gefitinib and erlotinib are being tested as treatments for various cancers, including nasopharyngeal cancer (Chua et al., 2008), gastric cancer (Dragovich et al., 2006), esophageal cancer (Wainberg et al., 2011), cervical cancer (Schilder et al., 2009), renal cell carcinoma (Gordon et al., 2009), and hepatocellular carcinoma (HCC) (Llovet and Hernandez-Gea, 2014). They have yet to receive approval for treatment and the results are not encouraging.

The most common side effects associated with gefitinib include acne rash, nausea, diarrhea, anorexia, stomatitis, and dehydration. These side effects were well tolerated by patients and the drug was withdrawn from patients who could not tolerate it. Overall, gefitinib is one of the more well-tolerated cytotoxic drugs (Forsythe and Faulkner, 2004).

Erlotinib has more severe adverse effects than gefitinib; however, like gefitinib, it is well tolerated. Dose reduction due to intolerance to side effects was more frequent with Erlotinib. According to reports, this drug caused significantly high rates of severe skin rashes, diarrhea, nausea, and vomiting, as well as stomatitis (Zhang et al., 2018).

#### Sunitinib (Sutent, SU11248)

Sunitinib is a first-generation VEGFR TKI and is a multi-target ATP competitive TKI. In various solid tumors, VEGFR is overexpressed and induces angiogenesis by binding to the vascular endothelium. Sunitinib is known to inhibit various tyrosine kinases, such as VEGFR2, PDGFRβ, KIT, RET, CSF1R, and FLT3 (O’Farrell et al., 2003; Faivre et al., 2007; Polyzos, 2008). In 2006, the FDA approved sunitinib for the treatment of metastatic RCC (mRCC) based on a phase III trial in which sunitinib exhibited superiority over IFN therapy. Sunitinib has also been approved for the treatment of imatinib-resistant gastrointestinal stromal tumors (GISTs). Furthermore, sunitinib was approved by the FDA in 2017 as an adjuvant therapy for adult patients at high risk of CCR after nephrectomy. In addition, in a phase III randomized trial, sunitinib was evaluated in patients who had nephrectomy and loco-regional RCC. Sunitinib patients exhibited a significant increase in disease-free survival compared with the placebo group (HR-0.76; 6.8 vs. 5.6 years, respectively [95% CI 0.59–0.98; *p* = 0.03]) (Ravaud et al., 2016).

In another landmark phase III trial, sunitinib was tested against interferon-α in patients with advanced metastatic RCC. Sunitinib patients showed significantly longer PFS compared with interferon patients (11 months vs. 5 months, respectively (HR-0.42 [95% CI 0.32–0.54; *p* < 0·0001]) (Motzer et al., 2007). Common side effects associated with sunitinib include grade 3 neutropenia, leukopenia, diarrhea, fatigue, nausea, hypertension, and hand-foot mouth syndrome (Demetri et al., 2006).

#### Lapatinib

Lapatinib is a next-generation inhibitor that also targets HER2 TK in addition to EGFR. Lapatinib was designed and developed to inhibit the action of HER2 TK in patients with HER2-positive breast cancer (Cameron and Stein, 2008). FDA approval was given to lapatinib in 2007 to treat HER2 patients with metastatic and advanced breast cancer. This approval was followed by a phase III open-label study that compared the use of lapatinib and capecitabine with capecitabine alone in HER2 patients with advanced metastatic breast cancer who had previously been treated with anthracycline, taxanes, and trastuzumab. With the combination therapy, the median time to progression increased significantly to 8.4 months from 4.4 months, without increasing the toxic effect or systematic cardiac effect (Geyer et al., 2006). In 2010, lapatinib was approved for the treatment of patients with HER2-positive advanced breast cancer in combination with letrozole as a first-line agent. This approval was based on the results of a double-blind randomized phase III trial in postmenopausal women with HER2-positive advanced breast cancer. PFS was significantly prolonged in the combination therapy group compared with the letrozole and placebo groups, and the total response rate was 28% vs. 15%, respectively. Additionally, the combination therapy group had a clinical advantage of 48%, compared with 29% for letrozole alone, and experienced diarrhea 68% of the time and a rash 46% of the time. The cardiac side effect observed in trastuzumab was not observed in the letrozole group (Schwartzberg et al., 2010).

#### Pazopanib

Pazopanib is a multi TKI. It is an inhibitor of VEGFR, PDGFR (A and B), c-Kit, and fibroblast growth factor receptor (FGFR) (van Geel et al., 2012). For metastatic RCC, pazopanib was approved by the FDA in 2009, and in 2012 it was approved by the FDA for the treatment of advanced-stage soft tissue sarcoma. A global multicenter placebo-controlled randomized phase III double-blind trial was conducted with patients with locally advanced and metastatic RCC. Pazopanib significantly improved PFS compared with the control group (9.2 months vs. 4.2 months, respectively), with a median duration response of more than 1 year (Sternberg et al., 2010). The FDA approved pazopanib as a first-line therapy for patients with metastatic RCC based on the findings of this study. By contrast, in a placebo-controlled phase III randomized trial, in which adjuvant sunitinib or sorafenib was tested in patients with high-risk non-metastatic RCC (ASSURE), pazopanib did not outperform the placebo (Motzer et al., 2017).

In a randomized controlled double-blind phase III trial that studied the preference of treatments in patients with metastatic RCC (PISCES), more patients preferred pazopanib (approximately 70%) than sunitinib (22%), whereas approximately 8% had no preference (Escudier et al., 2014). This proved that there was a clinical preference for pazopanib, which had the same therapeutical potentials but with a different profile of side effects. The side effects exhibited with pazopanib include diarrhea, change in hair color, and increased enzymes in the liver (Sternberg et al., 2010).

#### Ruxolitinib

A mutation in the JAK2 kinase domain is found in approximately 95% of patients with polycythemia vera (PV) and 50% of patients with essential thrombocytosis (ET) and myelofibrosis (MF), which causes pathogenesis at the molecular level (James et al., 2005; Kralovics et al., 2005). Ruxolitinib is a non-receptor TKI of JAK2 and Janus kinase. Several studies have tested the efficacy of ruxolitinib in all three myeloproliferative diseases. Ruxolitinib is approved for treating polycythemia vera (PV) patients who have hydroxyurea resistance or intolerance. Additionally, in 2011, ruxolitinib was approved for patients with intermediate to high-risk myelofibrosis (MF) and who were PV resistant or intolerant to hydroxyurea (2014). However, it has been noted that a significant number of patients receiving ruxolitinib lose their hair, experience a subpar response, or develop cytopenia, and this causes them to stop taking the medication after a few months. In fact, in some of the phase III trials, the discontinuation rate was 50% and 70% at 3 and 5 years, respectively. Some of the side effects reported for this drug include anemia, thrombocytopenia, and herpes zoster infection (Harrison et al., 2012; Verstovsek et al., 2012; Vannucchi et al., 2015).

#### Vemurafenib

Vemurafenib was the first BRAF kinase inhibitor approved (by the FDA in 2011) for the treatment of metastatic melanoma patients with the BRAF V600E mutation. A landmark phase III randomized control trial compared Vemurafenib (RO5185426) with dacarbazine in patients with BRAF V600E mutant metastatic melanoma who had not previously received any treatment. At 6 months, the OS in the vemurafenib group increased significantly to 84% (95% CI 78–89) compared with 64% in the dacarbazine group (95% CI 56–73, HR-0.37; 95% CI 0.26–0.55; *p* < 0.001), and the median PFS for vemurafenib was 5.3 months compared with 1.6 months for dacarbazine (Chapman et al., 2011). In the extended study, there was a significant increase in the median OS in the vemurafenib group (95% CI 12.0–15.2) compared with the dacarbazine group (13.6 months vs. 9.6 months, respectively; 95% CI 7.9–12.8; HR-0.70; 95% CI 0.57–0.87; *p* = 0.0008). The median PFS also increased significantly in the vemurafenib group (6.9 months; 95% CI 6.1–7.0) compared with the dacarbazine group (1.6 months; 95% CI 1.6–2.1; HR-0.38; 95% CI 0.32–0.46, *p* < 0.0001 (Chapman et al., 2011; McArthur et al., 2014).

### Type II inhibitors

#### Imatinib

Ciba-Geigy discovered imatinib while screening for platelet-derived growth factor receptor (PDGFR) kinase inhibitors. Interestingly, the compound CGP53,716 demonstrated significant activity against Abl-kinase; further modification of this compound to specifically target Bcr-Abl kinase resulted in the development and discovery of imatinib (ST571) (Wong and Witte, 2004). Owing to previous studies demonstrating its efficacy in patients for whom first-line therapy failed, imatinib was tested against standard therapy (interferon and cytarabine) in patients with previously untreated CML (Druker et al., 2001; Kantarjian et al., 2002). The FDA later approved imatinib as a first-line therapy for chronic CML. Additionally, it has since been approved for treating rare hematologic malignancies, as well as proto-oncogene c-Kit or tyrosine-protein kinase Kit-mutated GIST (Imatinib, 2008).

In a landmark phase III international multicenter crossover randomized study of interferon and STI571 (IRIS), imatinib was compared with a combination of interferon and cytarabine in patients with chronic CML. There was a significant increase in complete hematologic response in the imatinib group (95.3%; 95% CI 93.2–96.9) compared with the combination therapy group (interferon and cytarabine) (27.3%; 95% CI 6.0–61.0). Additionally, there was a major cytogenic response in the imatinib group (85.2%; 95% CI 81.9–88.0) compared with the combination group (22.1%; 95% CI 18.7–25.8). Although there was no significant difference in OS, it should be noted that around 89.2% (493 out of 553) patients, either discontinued or shifted to other group (O'Brien et al., 2003). Sixty-month follow-up showed that only 3% of the patients remained in the combination group. Furthermore, the follow-up showed a complete hematologic response of 98%, a cytogenic response of 92%, and an overall survival rate (OSR) of 89% (95% CI 86–92) in the imatinib group (Druker et al., 2006). Follow-up at 120 months showed that 48.3% of patients who were randomly assigned to the imatinib group completed the treatment regime assigned to them, whereas 1.3% completed the treatment regime in the interferon and cytarabine group. The OSR in the imatinib group was 83.3% (95% CI 80.1–86.6) (Hochhaus et al., 2017). Neutropenia, thrombocytopenia, anemia, elevated liver enzymes, and other drug-related adverse events were observed (Druker et al., 2006). In a 10-year follow-up study, no new additional side effects were observed (Hochhaus et al., 2017).

#### Nilotinib

Nilotinib is a second-generation Bcr-Abl kinase inhibitor that was developed to overcome the imatinib mutant. Nilotinib is 20-fold more efficacious than imatinib. An *in vitro* study of the inhibitory activity of nilotinib suggested that it was able to inhibit most of the 15 Bcr-Abl mutant resistant receptors (Weisberg et al., 2005). It was designed to be more efficacious than imatinib and with lesser side effects than its counterpart. Nilotinib was approved by the FDA as a first-line therapy for patients with chronic CML and for patients who develop resistance or are intolerant to imatinib. In a phase III multicenter randomized clinical trial (ENESTnd), the safety and efficacy of 300 mg BID and 400 mg BID of nilotinib was compared with 400 mg BID of imatinib in patients with advanced-stage CML. The 12-month study revealed that 300 mg BID of nilotinib resulted in a response rate (44%) double that of imatinib 400 mg BID (22%). The cytogenic response was 80% in the 300 mg BID nilotinib group and 65% in the imatinib group; a similar response was observed in a 24-month follow-up, which showed a twofold increase of 71% in the nilotinib group and 44% in the imatinib group. Complete cytogenic response was 26% in the nilotinib group and 10% in the imatinib group. However, although there was a lower rate of death related to CML, there was no improvement in OS in the nilotinib group (Saglio et al., 2010; Kantarjian et al., 2011). The molecular response in the 300 mg BID nilotinib group was significantly higher in the 60-month follow-up study (Hochhaus et al., 2016). Rashes, headaches, diarrhea, fluid retention, cardiovascular events, cytopenia, and biochemical abnormalities were all reported as side effects (Hochhaus et al., 2016).

#### Sorafenib

Sorafenib, like sunitinib, is a first-generation VEGFR TKI. It inhibits VEGFRs, PDGFRs, and Fms-related tyrosine kinase/Flk2/Stk-2-receptor (FLT3R). It was the first TKI to be approved for the treatment of metastatic RCC in 2005, and it is currently used as a second-line agent in the treatment of patients with metastatic RCC. Sorafenib was approved for HCC in 2007, and for metastatic thyroid cancer in 2013.

In a phase III randomized double-blind placebo-controlled trial of advanced RCC named TARGET (Approaches in Renal Cancer Global Evaluation Trial), sorafenib was compared with placebo. The results showed that sorafenib significantly improved median PFS (5.5 months vs. 2.8 months; HR-0.44, 95% CI 0.35 to 0.55; *p* < 0.01) (Escudier et al., 2007), but the efficacy and the safety evaluation did not show any benefits with respect to OS. However, when the post crossover placebo survival data were censored, there was a significant improvement in OS in the sorafenib group (17.8 vs. 14.3 in the control; HR-0.78, *p* < 0.029) (Escudier et al., 2009). Additionally, sorafenib was compared with a placebo in a multicenter phase III double-blind placebo trial of sorafenib in patients with advanced HCC (Hepatocellular Carcinoma Assessment Randomized Protocol [SHARP]) and was shown to significantly increase the median OS (7.9 months–10.7 months) (Llovet et al., 2008).

The efficacy of sorafenib was tested in patients with radioactive iodine-refractory locally advanced or metastatic differentiated thyroid cancer in the multicenter phase III randomized placebo-controlled trial Nexavar *versus* Placebo in Locally Advanced/Metastatic Radio-active Iodine Refractory Differentiated Thyroid Cancer (DECISION). The results showed that sorafenib was associated with a significantly longer PFS than the placebo group (10.8 months vs. 5.8 months; HR-0.59, 95% CI 0.45–0.76; *p* < 0.0001) (Brose et al., 2014). Side effects that are commonly encountered with this inhibitor include hypertension, fatigue, anemia, vomiting, and diarrhea (Escudier et al., 2007; Escudier et al., 2009; Brose et al., 2014).

#### Ponatinib (Iclusig, IY5511)

Owing to the steric hindrance caused by the bulky isoleucine residue at the 315 position, all previously produced Bcr-Abl kinases find it difficult to bind to this mutant T315I kinase. To overcome this steric hindrance, a computational and structure-based approach was used (Zhou et al., 2011b). Ponatinib was developed specifically to bind to T315I mutant kinases. The results of *in vitro* studies demonstrated that it was active against all mutant T315I Bcr-Abl kinases (O'Hare et al., 2009).

A significant response to ponatinib was observed in patients who did not respond to therapy, patients with a T315I mutation, and patients who exhibited refractory therapy to TKI with an undetectable mutation in Bcr-Abl (Cortes et al., 2012; Cortes et al., 2013). In 66% of patients with a T315I mutation, ponatinib was able to induce the cytogenic response, proving its clinical efficacy. However, in generalized CML-CP patients it did not show superior efficacy to previous TKIs. Therefore, this suggests that it may be used as first-line therapy in patients with a T315I mutation or otherwise just as a second-line therapy following the use of first- and second-generation TKIs (Cortes et al., 2012). The phase III trial for the evaluation of ponatinib vs imatinib in chronic myeloid leukemia (EPIC), designed to study the safety evaluation and clinical efficacy, was terminated due to previous reports of arterial thrombosis (Lipton et al., 2016). Therefore, the use of ponatinib in patients with newly diagnosed chronic myeloid lymphoma has not yet been established.

Ponatinib was compared with Allo-SCT in the Philadelphia Positive Acute Lymphoblastic Leukemia and Chronic Myeloid Leukemia Evaluation Trial (PACE). Ponatinib exhibited a significantly longer OSR at 24 and 48 months (24 months, 84% vs. 60.5%, *p* = 0.004; 48 months, 72.7% vs. 55.8%, *p* = 0.013; HR −0.37, 95% CI 0.16–0.84; *p* = 0.017) (Nicolini et al., 2017). To overcome the resistance of ponatinib, it is necessary to further understand the mechanism by which resistance occurs (Zabriskie et al., 2014; Wang et al., 2017; BCR-ABL1 compound mutations drive ponatinib, 2014). In 2014, the FDA narrowed the recommended use to ponatinib for adult patients with T315I-positive CML or ALL who had no other TKI options due to the risk of arterial embolism. Ponatinib side effects include rash (47%), abdominal pain (46%), dry skin (42%), thrombocytopenia (46%), and headache (43%). Additionally, constipation (41%), diarrhea, pyrexia, myocardial infarction, anemia, neutropenia, and pancytopenia have been reported. The arterial thrombotic event resulting from the treatment was observed by the onsite investigators in 2.2%, 0.7%, and 1.6% patients (Lipton et al., 2016). The most severe adverse effect observed in approximately 31% of the patients was arterial occlusive events (Cortes et al., 2013).

### Type III inhibitors

#### Trametinib and cobimetinib

##### Trametinib

Trametinib is an inhibitor of MEK and was developed to treat BRAF-mutated metastatic melanoma by targeting MEK. In 2013, the FDA approved trametinib for the treatment of BRAF V600E mutant metastatic melanoma, and in 2014 the FDA approved its use in combination with dabrafenib. This combination is currently being approved as a first-line treatment for BARF V600E mutant metastatic melanoma.

In a phase III randomized clinical trial (METRIC), the efficacy of trametinib vs. dacarbazine was tested in patients with advanced stage or metastatic BRAF V600 E/K mutant-positive melanoma. The results of the clinical trials showed that trametinib significantly increased the median PFS (4.8 months in the trametinib group and 1.5 months in the dacarbazine group). In the trametinib group, the HR for progression of disease or death was 0.45 (95% CI 0.33–0.63; *p* < 0.001) (Flaherty et al., 2012).

In another study, a double-blind phase III randomized control trial, a comparison was made between dabrafenib monotherapy and trametinib and dabrafenib combination therapy in patients with BRAF mutant melanoma (COMBI-d). The results of the primary analysis at 9 months follow-up showed a median PFS of 9.3 months for combination therapy and 8.8 months for dabrafenib monotherapy (HR-0.75; 95% CI 0.57–0.99, *p* = 0·0348) (Long et al., 2014). OS was 47% in the combination group vs., 58% in the monotherapy group (HR-0.71; 95% CI 0.55–0.92, *p* = 0·0107). The median OS was 25.1 months for the combination group vs. 18 months for the dabrafenib group (Long et al., 2014).

The results of a double-blind phase III randomized control trial (COMBI-v) of trametinib in combination with dabrafenib against vemurafenib alone were similar for each treatment in patients with unresectable or metastatic BRAF V600 E/K cutaneous melanoma. The results showed a significant increase in the OS and median PFS at 12 months in the combination group (72% vs. 65% for vemurafenib alone).

A phase III double-blind placebo controlled randomized trial of dabrafenib with trametinib, an MEK inhibitor, was carried out for adjuvant therapy for patients with high chances of BRAF V600 mutant-positive stage III melanoma after surgical resection (COMBI-AD). The result of the 2.8-year follow-up study showed that the estimated 3-year free survival rate in the combination group was 58% compared with 39% in the placebo control group (Long et al., 2015; Long et al., 2017a).

Some of the common side effects associated with trametinib include rash, diarrhea, and peripheral edema. Asymptomatic and reversible reduction in cardiac ejection fraction and toxic ocular effect were not frequent. It may be interesting to note that the secondary skin neoplasm observed in patients with the BRAF KI was not observed in patients who received a combination of MEK inhibitors (Flaherty et al., 2012).

##### Cobimetinib

The FDA approved the MEK inhibitor cobimetinib in combination with vemurafenib (a BRAF inhibitor) for the treatment of patients with mutant BRAF V600E metastatic melanoma after a phase III controlled randomized trial comparison study between vemurafenib alone and vemurafenib and cobimetinib in combination. In the study, the combination group showed a prolonged median PFS of 9.9 months compared with 6.2 months in the vemurafenib group (HR for death or disease progression was 0.51; 95% CI 0.39–0.68, *p* < 0.001) (Long et al., 2017a). The common side effects observed were pyrexia and elevated levels of liver enzymes (Larkin et al., 2014; Long et al., 2017a).

### Type IV inhibitors

#### Temsirolimus

The first mTOR inhibitor approved was rapamycin, also known as sirolimus. It is produced by *Streptomyces hygroscopicus* (Sehgal et al., 1975) and is an antifungal agent with poor pharmacokinetic properties. However, much of the research has been focused on the development of synthetic analogs of rapamycin that have a good pharmacokinetic profile suitable for therapy (Liu et al., 2009). These synthetic analogs, such as everolimus, temsirolimus, and radaforolimus, differ from sirolimus at the C-40-O position, resulting in superior pharmacokinetic and pharmacodynamic profiles, with suitable therapeutic potentials. These drugs are used in the treatment of solid tumors, such as RCC, breast cancer, pancreatic neuroendocrine tumors, and tuberous sclerosis complex (Awada et al., 2008).

Targeting of mTOR kinase may benefit the management of mRCC, and temsirolimus is one of the available inhibitors. Significant results from phase III trials led, in 2007, to FDA approval of temsirolimus as a single agent in poor rim RCC patients Bergmann et al. (2014). The results showed superior OS with temsirolimus compared with IFN-α or temsirolimus plus IFN-α. Additionally, temsirolimus showed benefits against clear cell carcinoma and papillary RCC.

### Type V inhibitors

#### Lenvatinib

Lenvatinib is a TKI that inhibits VEGFR and various other FGFRs, such as FGFR1, FGFR2, FGFR3, FGFR4, PDGFRs, RET, and c-Kit (Okamoto et al., 2015; Kannaiyan and Mahadevan, 2018). It was approved for the treatment of radioactive iodine-refractory differentiated thyroid cancer by the FDA in 2015, and in 2016, the FDA approved it as a second-line therapy in combination with everolimus for treating metastatic RCC after anti-angiogenic therapy.

In a phase II randomized open-label multicenter trial, lenvatinib was tested as a second-line therapy for patients with metastatic RCC who had undergone anti-angiogenic therapy; lenvatinib was compared with everolimus for its efficacy, as well as the combination of everolimus and lenvatinib. The combination of lenvatinib and everolimus increased the median PFS to 14.6 months compared with 5.5 months for everolimus alone, whereas a significant improvement in the median PFS (7.4 months) was observed in the lenvatinib monotherapy group compared with the everolimus group (Motzer et al., 2015).

In a phase III randomized double-blind trial, lenvetanib was investigated against the placebo in patients with progressive thyroid cancer refractive to radioactive iodine. Lenvatinib showed a median PFS of 18.3 months compared with 3.6 months with the placebo (HR for progression was 0.21) (Schlumberger et al., 2015). The side effects that were observed in patients included hypertension, fatigue, nausea, vomiting, lack of appetite, diarrhea, and hand-foot syndrome (Motzer et al., 2015).

### Type VI inhibitors

#### Afatinib (Gilotrif, BIBW2992)

Afatinib is an orally available second-generation EGFR TKI. The second-generation EGFR TKIs were designed to target other members of ErbB group, including HER2. They not only target the T790M mutation of EGFR, but other EGFR-activating mutations and wild-type EFGR (Wind et al., 2017).

Afatinib is an irreversible inhibitor of the ERBB family that includes HER1 (EGFR), HER2, and HER4. Compared with chemotherapy, afatinib was effective in prolonging PFS (11.1 months vs. 6.9 months, respectively) (Yang et al., 2013). Like erlotinib, afatinib also increases PFS significantly by 18% (19% OS).

In NSCLC patients for whom chemotherapy failed, afatinib, like erolitinib, significantly increased PFS by 18% and OS by 19%, and it was better in terms of disease control rate (51% vs. 40%). Subsequently, the FDA approved the use of afatinib as a first-line therapy in patients with NSCLC.

In the phase III randomized clinical trial LUX-Lung 8, afatinib was compared with erolitinib in patients with advanced squamous cell carcinoma as a second-line therapy. Subsequently, afatinib was approved as a second-line treatment due to the findings of the study (Soria et al., 2015). Diarrhea, rashes or pimples on the skin, and weariness are among the side effects frequently reported with afatinib (Paz-Ares et al., 2017).

#### Dacomitinib (PF299804)

Dacomitinib is an oral EGFR KI that irreversibly inhibits ERBB family members; it covalently binds to the receptor and blocks the downstream signaling pathway. It is more effective than the first-generation EGFR TKIs against all wild-type EGFRs and EGFR with Del19 or L858R mutations, with broad activity against multiple ErbB receptors, such as EGFR, HER2, and HER4. On 27 September 2018, the FDA approved dacomitinib (Vizimpro, Pfizer) as a first-line therapy for patients with metastatic NSCLC or EGFR with Del19 or exon 21 L858R mutations, as detected by an FDA-approved test (Reckamp et al., 2014; Wu et al., 2017a). The dose-limiting toxicity exhibited by second-generation EGFR TKIs is mainly exhibited in the skin and gastrointestinal tract because they also inhibit wild-type EGFRs (Sullivan and Planchard, 2016).

#### Neratinib

Neratinib is a TKI that targets all EGFRs and also inhibits EGFR tyrosine kinase. It is approved by the FDA as an adjuvant therapy for patients with early-stage breast cancer who test positive for HER2 after trastuzumab adjuvant therapy. This approval was based on the findings of a phase III randomized clinical trial that looked at the efficacy and safety of neratinib after adjuvant therapy with neoadjuvant and trastuzumab in patients with HER2-positive early-stage (I-III) breast cancer. Some of the side effects observed included diarrhea, nausea, and vomiting (Martin et al., 2017).

**FIGURE 1:**
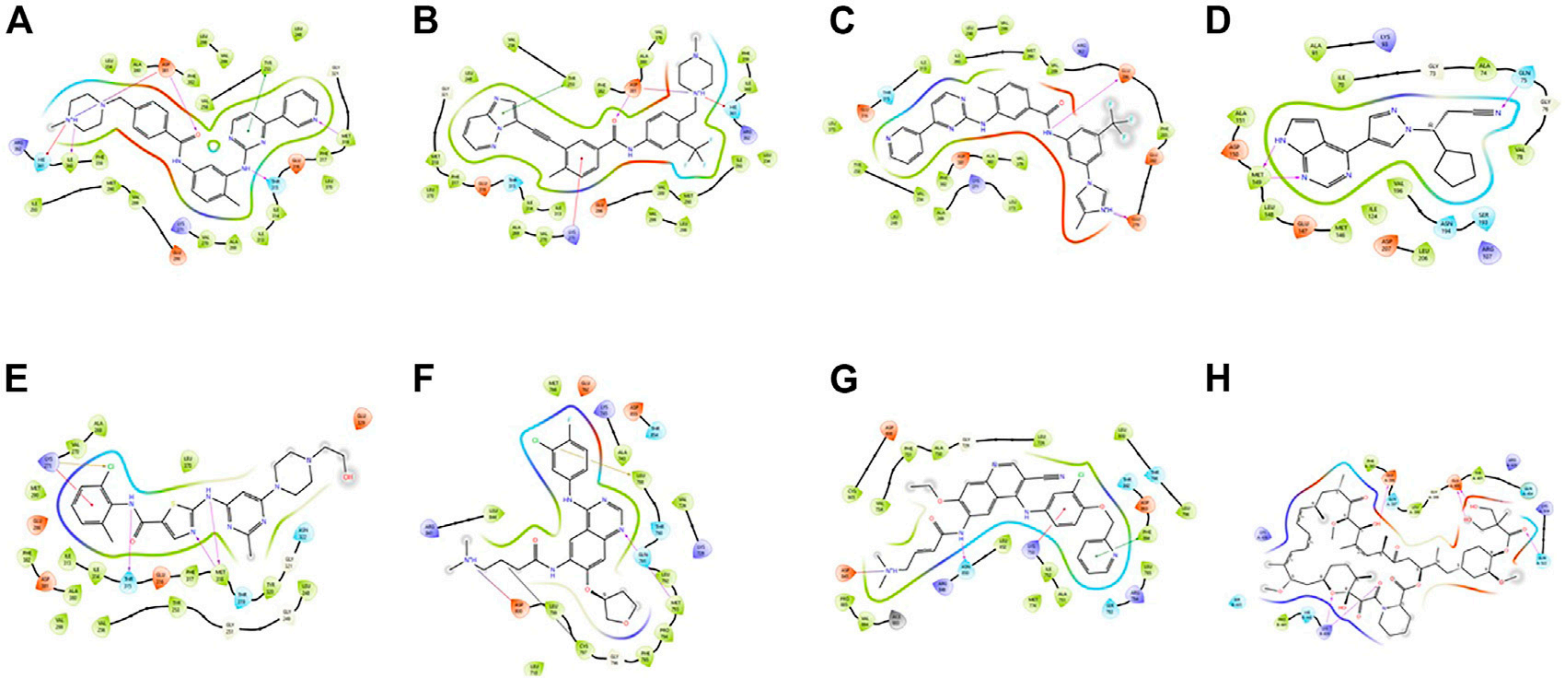
Docking poses of different proteins with drugs.**(A)** Imatinib. **(B)** Ponatib. **(C)** Nilotinib docked to human Abl kinase (2HYY). **(D)** Ruxolitinib dock to c-Jun N-terminal kinase (2P33). **(E)** Dasatinib docked to activated ABL kinase domain (2GQG). **(F)** Afatinib with EGFR kinase (4G5J). **(G)** Neratilib docked to HER2(3RCD). **(H)** Temsirolimus with human serum albumin (1AO6).

These small molecules interact with different receptors in various proteins. The interaction of these TKIs has been well studied using various molecular modeling software. Figure 1 shows one of the options in which molecular modeling and docking studies are carried out using Schrodinger software (Maestro 12.1, United States). In this review, these molecules have been redocked.

## Conclusion

The success of imatinib has led to the development of various KIs; currently (as of 29 October 2021) there are approximately 68 FDA-approved KIs in clinical use (http://www.brimr.org/PKI/PKIs.htm). All of these KIs are delivered orally, with a few exceptions, such as temsirolimus and trilaciclib, and netarsudil, which is available as an ophthalmic solution for eye drops. The development of KIs in cancer has led to a new approach in this regard. It may be noted that they are generally less toxic and have more chemotherapeutic potential than conventional chemotherapy. However, like conventional therapy, KIs have some limitations, such as the development of resistance, and some adverse effects.

Resistance developed during treatment can be addressed through the clonal expansion of cells that do not respond to specific kinase or secondary kinase mutations that render the kinase inhibitor ineffective. Understanding the resistance mechanism has resulted in the design and development of new KIs or combination therapies. For instance, development of the new Bcr-Abl KI ponatinib targeted the T315I mutant gene that was resistance to first- and second-generation Bcr-Abl inhibitors. Understanding the mechanism of resistance of EGFR T790M to the TKI of EGFR has led to the development of osimertinib, which targets the mutant gene of EGFR T790M. Combination therapy is another option for overcoming resistance; for example, understanding the mechanism of the MEK inhibitor that causes reactivation of the MAPK pathway, which in turn activates MEK towards the resistance of the BRAF inhibitors (in melanoma), prompted the use of combination therapy consisting of BRAF and MEK inhibitors, which resulted in better clinical outcomes than single therapy.

One of the main concerns associated with the use of KIs is adverse effects. As mentioned above, owing to the risk of arterial embolism, the FDA had to narrow down the recommended use of ponatinib. This issue can be addressed by choosing more specific KIs. Additionally, understanding the mechanisms of side effects may help to lessen their effect.

The remaining option for overcoming the above concerns is to continuously and thoroughly screen the patient’s condition, plasma concentration, and personalized treatment approach. Even if the stage and the cancer are the same, there may be differences depending on the patient’s status and tumor, hence a personalized treatment regime may be one of the best approaches.

Owing to the success of KIs, there is a rise in the design and development of new small molecule inhibitors. The use of new technologies, such as mass spectrometry-based proteomics, may help facilitate the development of more specific target-oriented inhibitors.
